# Demonstrating the Super-Recognizer advantage for law enforcement

**DOI:** 10.1016/j.isci.2026.116456

**Published:** 2026-07-09

**Authors:** Meike Ramon, Matthew J. Vowels

**Affiliations:** 1 Applied Face Cognition Lab, Business School, Bern University of Applied Sciences, Bern, Switzerland; 2 Association for Independent Research, Zurich, Switzerland; 3 Department of Cognitive Science, University of Malta, Msida, Malta; 4 Institute of Psychology, University of Lausanne, Lausanne, Switzerland; 5 The Sense, CHUV, Lausanne, Switzerland

**Keywords:** cognitive neuroscience, interdisciplinary application studies, psychology

## Abstract

So-called Super-Recognizers (SRs)—individuals with exceptional face recognition ability—are increasingly being deployed by international police agencies. Currently, however, empirical evidence supporting their utility for law enforcement stems from a single study based on a small forensic stimulus set and a predominantly civilian sample. Here, on the basis of data collected from within the entire cohort of ∼18,000 Berlin Police officers employed in 2021, we report the performance of officers identified as SRs, using previously published lab-based criteria under operationally relevant, highly challenging 1:*n* facial identity matching conditions via the Berlin Test for Super-Recognizer Identification (beSure®)—the only existing tool that uses authentic forensic material. As a group, lab-identified SRs exhibited increased proficiency across a range of forensically relevant tasks. However, lab-based SR selection methods showed limited sensitivity at the individual level. These findings support the utility of lab-based SR identification while emphasizing the value of bespoke, professionally relevant assessment for deployment within law enforcement.

## Highlights

• Lab-identified super-recognizers (SRs) outperform controls on authentic forensic material
• Group-level SR advantage holds across five forensic identity matching subtests
• Lab criteria show limited sensitivity for individual-level SR identification
• Bespoke forensic tools complement lab-based super-recognizer selection

## Graphical abstract

**Figure:**
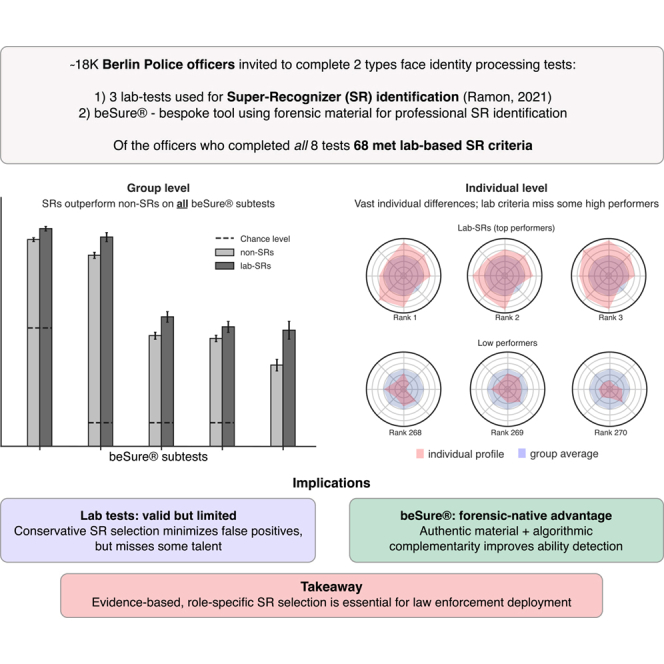

## Introduction

### Super-Recognizers: From lab discovery to real-world deployment

The term “Super-Recognizer” (SR) was originally introduced by Russell et al.,^1^ who described a handful of participants excelling at face matching, recognition, and identification. These SRs were discovered by happenstance while scientists were developing tests to identify individuals with impaired facial identity processing (FIP). Since then, despite an increasing number of scientific publications, the research community has not been able to develop a consensus on what actually makes a SR and how they should be identified (cf.^2^^,^^3^^,^^4^^,^^5^). Consequently, there are more open questions around this novel phenomenon, which remain to be answered definitively.

In the media, SRs have been portrayed as infallible superheroes “who never forget a face.” In actuality, there is currently no definitive information regarding *how much better* SRs actually are relative to average people. Individual SRs’ reported performance may differ largely due to the variability in tasks implemented, but also because of their unique, idiosyncratic performance profiles (as discussed by^6^^,^^7^^,^^8^). Crucially, the degree to which such idiosyncrasies among SRs will be uncovered also depends on the analytical approaches adopted (i.e., univariate group-level comparisons vs. in-depth single-case descriptions). Moreover, the question of how well SRs can be expected to perform relative to facial recognition technology (FRT) remains unanswered. Previous studies suggest that average observers’ performance may be enhanced or negatively impacted or biased via algorithmic assistance.^9^^,^^10^^,^^11^^,^^12^^,^^13^^,^^14^ How SRs—as a group or at the individual level—would perform under algorithmically assisted conditions remains largely uninvestigated.

Notwithstanding our limited knowledge of the phenomenon of SRs, it has received a surge in interest, both scientifically and in terms of their deployment in law enforcement, specifically in policing. To date, SRs have been integrated into a range of law-enforcement and security operations internationally. SRs are employed in tasks where reliable human FIP remains critical, such as reviewing CCTV footage, monitoring surveillance feeds, and verifying identities in investigative contexts or public events. A number of police agencies across Europe have formally implemented programs to identify SRs to support operations. For example, SRs identified professionally^15^ have been deployed in the context of the UEFA European Football Championship and investigations into the Berlin New Year’s riots. In general, SRs’ deployment is particularly valuable in scenarios where the use of FRT is not possible due to technical limitations or legal considerations.^16^

The increasing interest in SRs from international law enforcement agencies can be attributed to a combination of factors. Devices enabling the acquisition, processing, and global proliferation of digital material are ubiquitous. This makes investigations of crimes committed in public spaces increasingly complex: law enforcement professionals are challenged with growing volumes of to-be-processed, and often low-quality data.^2^^,^^3^ While countries differ in their use and acceptance of FRT within law enforcement, e.g., for forensic investigations,^16^ the final decision is made by humans.^17^ Thus, the (desired) deployment of SRs reflects the recognition that, despite advances in computer vision, highly skilled human observers will continue to play a vital role in operational decision-making and evidentiary review.

Deploying the most capable humans for the most critical professional roles seems intuitive and plausible. The growing interest in SR identification and deployment, therefore, could be considered a logical consequence of their discovery. However, currently, caution is warranted at least for two reasons, which we discuss in the following sections: First, existing SR diagnostic approaches vary highly. Second, empirical evidence for the *de facto* value of SR deployment in law enforcement, i.e., when *forensic* material is concerned, stems from a single, recent study.^7^

### Who qualifies as an SR?

Intuitively, given the use of a singular term to describe a particular group of individuals, one might expect SRs to constitute a homogeneous group of individuals with comparable characteristics. However, in reality, there is a large degree of heterogeneity. This heterogeneity likely reflects both genuine inter-individual differences between human observers in general, as well as diagnostic approaches adopted in the SR literature, which are outlined in Box 1. Below, we discuss both aspects, the accurate understanding of which is crucial for successful fusion of humans’ and machines’ abilities across various settings, especially those with security and legal implications.

Box 1 **Super-Recognizer diagnostics: Varied approaches**

Studies reporting SRs differ vastly in terms of their diagnostic approaches, which can fall into one of three categories depending on the (number of) tests used.

• **Single/same test**: One group of studies^6^^,^^18^^,^^19^^,^^20^^,^^21^ used the same test and criterion for SR identification. Specifically, the authors used the long version of the Cambridge Face Memory Test (CFMT+),^1^ with a (conservative) cutoff determined based on normative data collected from a large cohort of young British adults. This test involves 3-alternative forced choice recognition of 6 experimentally learned male Caucasian identities, thus probing face *memory.* Thus, with this approach, it is possible that individuals with superior face *perception* abilities remain undetected.
• **Single/any test**: Other studies have adopted a more indiscriminate approach. For instance, Phillips et al.^8^ reported SRs as individuals scoring “above average on any previously reported test in the SR literature.” Their accepted tests included the Glasgow Face Matching Test (GFMT^22^), which comprises 40 trials soliciting binary same/different identity decisions for high-resolution image pairs. Note that the GFMT lacks sensitivity, as also documented even by its developers who reported “normal test performance” in developmental prosopagnosia.^23^
• **Multiple tests**: The seminal work by Russell et al.^1^ reported SRs’ performance superiority across a fixed set of FIP tests, i.e. a *selection of tests* probing face *discrimination*, *recognition*, *and identification*. Note that even the same researcher groups have adopted different approaches. For example, Davis and colleagues applied a single/same test approach in one study,^24^ while reportedly using multiple tests (including tests lacking scientifically published procedures and normative data) elsewhere.^25^ Departing from their original single/same test approach (see above), Bate et al.^4^ recommended the use of at least two established tasks for SR screening, however, focusing narrowly on *memory*. The first formal SR diagnostic framework developed to mirror Russell and colleagues’ seminal report^1^ was proposed by Ramon.^5^ It involves three established FIP tests, which collectively assess face perception and face memory. As such, this latter approach is more flexible and appropriate for various applied policing settings, where image comparison - which does not rely on face memory - is critical. Critically, Ramon’s framework^5^ is the only one that has been validated with forensic perpetrator identification using authentic police material.^7^

One of the first published SR studies^6^ provided an in-depth neuropsychological investigation into six individuals, who had been identified as SRs based on their above average Cambridge Face Memory Test (CFMT+)^1^ performance, i.e., using a single test for SR identification (see Box 1). The authors reported that “while superior face-processing skills were restricted to face memory in three of the SRs, enhancements to facial identity perception were observed in the others.” This mirrors findings from neurotypical, i.e. average observers who also exhibited highly varied performance behavioral and oculomotor profiles, independent of their FIP ability.^26^^,^^27^^,^^28^

More recent studies into SRs identified using a different diagnostic approach involving multiple tests assessing both face perception and memory^5^ also support the notion of inter-individual differences across the entire FIP ability spectrum. For instance, Nador et al.^29^^,^^30^ conducted a number of psychophysical experiments to assess individuals’ sensitivity to spatial frequency information processing and the degree to which learned representations are image dependent or viewpoint invariant. These authors reported highly varied performance profiles, *both among controls and SRs*. Collectively, the aforementioned studies point out two important aspects. First, neither neurotypical nor SR individuals should by default be considered as monoliths. Second, measures used to assess even the same ability can differ largely in terms of their procedures (e.g., experimental design or stimuli) and task difficulty.

Crucially, methodological differences between means of assessment result in substantial psychometric differences between existing tests.^27^^,^^28^^,^^31^^,^^32^ A recent systematic investigation into numerous FIP tests among neurotypical observers reported weak inter-test correlations, low internal consistency, and at best moderate re-test reliability.^27^ These findings are particularly problematic considering the lack of consensus on how to identify SRs (cf.^1^^,^^2^^,^^4^^,^^5^), alongside law enforcement’s increasing interested in their operational deployment. To date researchers’ diagnostic approaches used to identify SRs vary largely, both across, and even *within* studies (see Box 1). To exemplify this with two extreme cases, Russell et al.^1^ described SRs as individuals who excelled across the same three tests (measuring distinct FIP sub-processes), while Phillips et al.^8^ grouped together individuals who could have excelled at “any given” FIP test reported in the literature. From a practitioner perspective, the abilities of individuals identified (in whichever way) as SRs in the lab would need to be confirmed under *real-life professional* scenarios.

### What’s missing: Putting SRs to the real-life test

At minimum, given the interest in deploying SRs in law enforcement, one would want to ensure that superiority assessed in the lab translates to real-world conditions. This is critical considering SRs’ conceivable positive potential as well as the negative implications of deploying individuals falsely considered as SRs - both for public safety and societal trust in institutions. As scientists, despite our best efforts, we cannot assume that our procedures are a *de facto* good representation of the varied and challenging conditions characteristic of practitioners’ professional tasks.^2^^,^^33^ That is, SRs identified using (whichever) lab-developed procedures would have to be put to the test under the diverse scenarios they are sought to be deployed in professionally.

Lab-based SR status may not transfer to operational roles for a number of reasons. The main one concerns the various differences between forensic materials and those developed by researchers. Even if researchers were aware of all possible, professionally critical real-life variations in forensic traces, their comprehensive experimental implementation may simply not be feasible.

Indeed, even practitioner-researchers’ attempts to implement experimental procedures that mirror operationally relevant scenarios may fail or fall short. For instance, Thielgen et al.^34^ aimed to mimic real-life CCTV scenarios and compare performance for this material with standardized FIP test performance. To this end, they created 11 videos, which unfortunately involved an extremely limited number of target actors (nine individuals) who appeared repeatedly in the recordings taken in public spaces. Clearly, this limited number of *staged* scenarios poses a number of issues, and the real-life validity of this practitioner-developed setup is, at best, extremely limited. As stated by Moreton et al.,^33^ if a person exhibits “superior performance on a laboratory task testing one element of face perception cognition [it] does not mean they are the best person to carry out a specific operational role that will involve a multitude of other requirements. […] By understanding what tasks professional practitioners carry out, future SR tests can be tailored to more appropriately meet the requirements of these tasks.”

Despite the concerns regarding ecological validity of FIP abilities measured in the lab and SRs’ increasing deployment in security critical operational settings, their actual potential for real-life operational settings remains underinvestigated. Existing empirical studies have demonstrated that, as a group, people who were identified as SRs through some form of lab-based procedure tend to excel at “other lab tests.”^35^^,^^36^ However, how individual SRs perform across *varied professionally relevant tasks and given different material* remains unknown.^33^ Put simply, because SRs had (until recently, as discussed below) not been systematically evaluated in real-life scenarios, it remained unclear whether SR deployment provides a net value for applied settings, specifically law enforcement and policing.

To our knowledge, only one single recent study provides empirical evidence suggesting an advantage of lab-identified SRs in law enforcement. Mayer and Ramon^7^ reported that SRs identified according to the aforementioned recently proposed multi-test diagnostic framework^5^ outperformed controls at CCTV footage-based forensic perpetrator identification. Moreover, SRs achieved “more correct perpetrator identifications, the better their performance across lab tests”. Notwithstanding its importance, this study was limited in terms of two critical aspects. First, the amount of forensic material provided by the police was extremely constrained; each participant viewed four CCTV sequences and performed five identity decisions in mugshot line-ups. Second, the majority of SRs reported were civilians, i.e., not police officers, whose impressions could have motivated further legal actions. Thus, to date, barring the recently published study,^7^ reports of SRs’ operational successes remain purely anecdotal. Moreover, and crucially, whether SRs could improve operational proficiency in addition to FRT for processing of *authentic forensic material*^16^ has never been investigated. Both aspects are relevant to determine the currently unknown degree to which SRs’ abilities mirror or could complement performance achievable via automatic FRT.

This study, involving a truly unique dataset, addresses two crucial knowledge gaps that have been and remain difficult to overcome: (1) systematic investigation of law enforcement professionals’ performance across varied settings including lab-based procedures *and* professional scenarios using authentic forensic material; and (2) assessment of FIP in a large cohort sampled from the entire body of officers of a state police agency, i.e. law enforcement practitioners. Based on data from a representative population that is typically under-represented in scientific research, we provide unprecedented, in-depth insights into professionaly relevant FIP performance at the *individual police officer level*. Adopting univariate, multivariate, and data-driven approaches, we describe relationships between two approaches that have been used to identify SRs for law enforcement: the aforementioned multi-test, lab-based SR diagnostic framework^5^ and the Berlin Test for Super-Recognizer Identification (beSure®),^15^ which represents the only professionally designed FIP ability assessment tool that uses authentic police material. On the basis of the idiosyncratic multi-dimensional ability profiles observed among the volunteering Berlin Police officers, we develop operationally relevant recommendations for law enforcement for professional identification and deployment of SRs.

## Results

**Figure 1:**
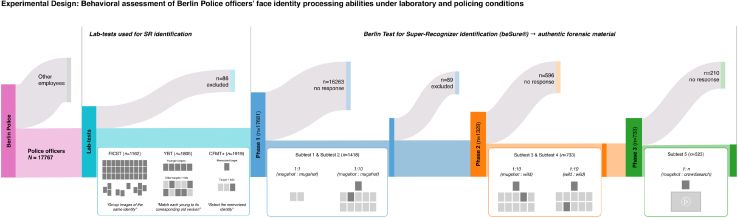
Experimental Design Illustrated here is the process of behavioral testing of Berlin Police officers via lab tests used for Super-Recognizer identification according to Ramon^5^ and subtests of beSure®. Note: All Berlin Police employee samples are illustrated in log scale.

**Figure 2:**
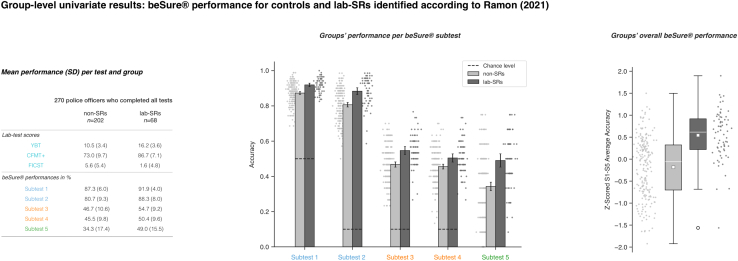
Results: Super-Recognizers vs. non-Super-Recognizers Performance of observers classified as SRs and non-SRs across all beSure® subtests. The table (left) provides descriptive statistics for the SR and non-SR groups per individual test. The bar chart (middle) indicates mean and 95% confidence intervals of the mean. The box-and-whisker plot (right) indicates interquartile range (box), mean (square), median (white line), and whiskers extending to the most extreme data point within 1.5× the IQR of the box edges, and outlier (circle).

Figure 1 illustrates the testing procedure, timeline and number of participants who volunteered at each stage (for details see STAR Methods). Given our goal of comparing performance on lab tests used for SR identification^5^ to that measured with beSure®,^15^ we conservatively restricted our analyses to data from only those participants who completed *all* eight subtests (*n*= 270). Of these, 68 met the previously proposed criteria for lab-based SR identification.^5^ Average lab and beSure® subtest scores are provided in Figure 2 for participants considered in the analyses reported below.

To examine the relationship between lab tests proposed for SR identification^5^ and those developed for professional SR identification within the Berlin Police, i.e., beSure®,^15^ we adopted a three-step analysis approach. First, we assessed whether—as a group—lab-identified SRs would perform significantly better on beSure®—both overall, as well as per subtest individually (univariate analysis). Second, we explored individual-level patterns by analyzing full performance profiles across all subtests to uncover relationships potentially obscured in the univariate analyses. This allowed us to evaluate, among police officers, whether those who met criteria proposed for SR identification and those who did not^5^ exhibited comparable or distinct performance profiles. Third, we used a combination of clustering and machine learning prediction to identify clusters and to see whether the SR group labels are predictable from their beSure® test results. Using multiple complementary analyses provided a form of triangulation, allowing us to assess the consistency of findings and to determine whether the results were sensitive to the analytical method adopted.

### Group-level univariate analyses

To investigate the group effects visualized in Figure 2, we performed a repeated-measures ANOVA with *group* (SRs vs. non-SRs) as a between-participant factor and *beSure*® *subtest* (S1–S5) as a within-participant factor, with a Greenhouse-Geisser correction (*ϵ* ≈ 0.656) applied to the within-participant effects due to violation of sphericity (Mauchly’s test; *W* = 0.367, *p* < 0.0001). First, there was a significant main effect of *group* $\left(F\left(1,268\right)=61.26,p<.0001,\eta_{G}^{2}=.091\right)$, indicating that SR participants generally outperformed non-SR participants across the beSure® subtests. Second, we observed a significant main effect of *beSure® subtest* $\left(F_{\text{GG}}\left(2.62,703.04\right)=1209.11,p<.0001,\eta_{G}^{2}=.717\right)$, confirming that task difficulty varied significantly across the subtests. Finally, the *group* × *beSure® subtest* interaction was significant $\left(F_{\text{GG}}\left(2.62,703.04\right)=10.04,p<.0001,\eta_{G}^{2}=.021\right)$, indicating that the performance difference between SR and non-SR groups depended on the specific beSure® subtest. Finally, Bonferroni-corrected post-hoc comparisons revealed that SRs significantly outperformed non-SRs on all subtests (S1, *t*(987) = 3.007, *p* = 0.0027, *d* = 0.42; S2, *t*(987) = 4.998, *p* < 0.0001, *d* = 0.70; S3, *t*(987) = 5.165, *p* < 0.0001, *d* = 0.72; S4, *t*(987) = 3.185, *p* = 0.0015, *d* = 0.45; and S5, *t*(987) = 9.587, *p* < 0.0001, *d* = 1.34).

### Multivariate analyses of individual profiles

**Figure 3:**
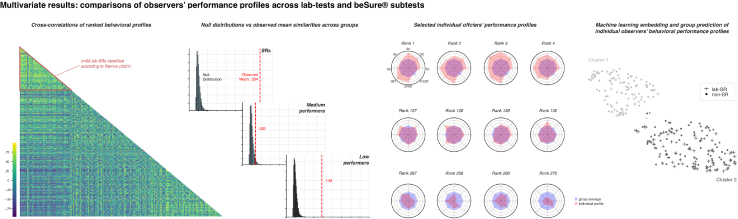
Results: Individual behavioral profiles and group status prediction Observers’ overall ability level and relation to their performance profiles across all eight behavioral measures of face identity processing.

To characterize performance differences in greater detail considering all available dimensions (i.e., all eight scores per officer), we investigated differences across observer *profiles* that were not considered in the aforementioned univariate (group) analyses. The performance similarity matrix (PSM), shown in Figure 3 (left) visualizes for all participants (ranked according to their overall performance), the outcome of pairwise correlations between their standardized (z-scored) test scores. Each cell (color) represents the overall similarity of observers’ performance profiles across the eight performance measures (three lab tests and five beSure® subtests). For example, two participants with high lab test and low beSure® performance would share the same across-test performance variation, thus resulting in a high pairwise correlation (i.e., a similar performance profile).

To explore the overall performance similarity as a function of participant group, we computed the average of all SRs’ pairwise correlations and compared this against the average pairwise correlations for non-SRs. Specifically, we extracted and averaged SRs’ PSM results, which are delineated in red in the PSM shown in Figure 3 (left). Owing to the highly interdependent/structured nature of the matrix, it is ill-advised to use simple group comparisons to infer whether the groups are significantly different from one another. Instead, we created a “surrogate” null distribution by randomly permuting a randomly sampled submatrix with the same size as the comparison group 10,000 times and comparing the distribution of means for these permuted matrices against the mean of the similarity submatrix for the comparison group. In order to investigate differences in profile/similarity as a function of ability (rank), we ranked the similarity score samples according to contributing participants’ overall performance (i.e., the average across all z-scored tests scores) and extracted the average similarity score for each participant drawn from the 68 medium- and 68 low-performing non-SRs (i.e., observers ranked from 102 to 169 and 203 to 270, respectively), in order to compare against the null. As such, we arrived at three comparison groups comprising 68 observers each: SRs, medium-performers, and low-performers (applying a Bonferroni-corrected *α* = 0.0167 to account for multiple comparisons).

In all three cases, the bootstrapped/permuted null distributions were found to have an expected value very close to zero (M < 0.0001 in all cases). The average performance similarity for the SRs was found to be significantly different from the associated randomly permuted matrices (M = 0.334, *p* < 0.0001). In contrast, following the application of Bonferroni correction, we found that medium-performing participants were not significantly different from the null (M = 0.020, *p* = 0.036), whilst low-performers were significantly different (M = 0.143, *p* < 0.0001). This pattern reflects higher inter-observer performance similarity at both ends of the performance-ranked continuum: the profiles among the highest-ranking observers (i.e., SRs), as well as lowest-ranking observers are relatively more similar to one another. Profiles of observers occupying the medium ranks, on the other hand, had a mean similarity that was closer to the expected value of the null distribution. The analysis of performance similarity across the three groups considered the “average similarity” among the 68 observers of a given group, relative to the similarity observed for a random selection of 68 participants. As such, this analysis cannot provide a full representation of the nature of the observed (dis)similarity. To exemplify this, consider the radar plots in Figure 3, which helps visualize individual observers’ performance profiles across all eight available measures. Within each of the three groups, we found that individual observers’ profiles (i.e., “shape” of the orange areas) deviate from the group’s average profile (purple-shaded area). This indicates that observers exhibit idiosyncratic profiles, *in general and independently of their ability* (inferred via measured performance levels).

### Machine learning embedding and group prediction

In Figure 3 (right) we visualize a two-dimensional embedding of each participant’s beSure® accuracy profile. Each point corresponds to one participant represented by a five-dimensional vector of subtest accuracy scores on tests S1–S5. The vertical and horizontal axes are abstract coordinates produced by the uniform manifold approximation and projection (UMAP)^37^ algorithm, which places participants closer together when their across-subtest accuracy profiles are more similar, and farther apart when they are less similar. Points are colored by the hierarchical density-based spatial clustering of applications with noise process (HDBSCAN)^38^ applied to the same accuracy profiles, and marker shape indicates SRs versus non-SRs. These techniques are highly flexible and data adaptive and can be used to uncover underlying structure in multi-dimensional data. As such, the purpose of this projection is exploratory—to assess whether participants form natural groupings based on their overall beSure® performance pattern, and to check whether these groupings align with lab-based SR status following Ramon's diagnostic framework.^5^ The results highlight two primary clusters (light and dark gray coloring) and the boundary between these groups are clearly correlated with SR status: most SRs are in the bottom-right, dark gray set of points.

Finally, and taking a less exploratory approach than embedding and clustering, in order to test the capacity of the beSure® subtest results for separating SRs from non-SRs, we used a random forest (RF) machine learning algorithm^39^ as included in the scikit-learn Python library.^40^ A RF is a type of decision tree that learns by training on bootstrapped subsamples of the data (subsamples of both variables and participants) in order to mitigate overfitting. We used the default settings, which have been shown to work well across a range of applications.^41^ We implemented a leave-one-out cross-validation strategy, where all participants’ data but one randomly selected participant are used for training, and a prediction is made for the unseen participant. The process is repeated until predictions have been accumulated for all participants (each time the RF is retrained from zero), resulting in a set of predictions for the participants the RF does not see during training. This gives a reliable estimation of the performance of the RF. We used balanced accuracy, which accounts for group size imbalance, to evaluate the final degree to which SR status is predictable from the beSure® subtests.

It is worth noting that the purpose of this supervised analysis is convergent validity rather than definition of police SRs. Specifically, we ask whether the “across-subtest” beSure®^15^ accuracy profiles contain information that aligns with an external criterion label obtained from the lab test battery.^5^ The final balanced accuracy was found to be 0.65, i.e., above chance level (0.5). These results suggest that successful prediction of the SR labels using the results of the beSure® subtests is indeed possible. Note that this analysis does not claim that beSure® alone identifies SRs, but only that its profiles predict lab SR status above chance.

## Discussion

SRs—individuals with exceptional FIP ability—were first identified incidentally in a laboratory context^1^ and have since attracted widespread scientific and applied interest. Despite increasing deployment in operational settings and numerous anecdotal reports of their successes, systematic investigations into SRs’ utility in professional law enforcement contexts remain scarce. To date, only one study has reported that SRs outperform controls in perpetrator identification using authentic police materials.^7^ However, the majority of participants in that study were civilians, and the number of identity decisions per observer was extremely limited (four videos requiring five identity decisions) due to the sensitive nature of the forensic material used. Consequently, whether lab-identified SRs can reliably enhance operational outcomes in professional law-enforcement settings remains an open question.

Addressing this gap, the present study provides an unprecedented, large-scale validation of the utility of the recently proposed first formal diagnostic lab-based framework for SR identification^5^ that aligns with the seminal SR report.^1^ We report results obtained from lab-identified SRs and non-SRs under professionally relevant conditions pertaining to FIP in law enforcement. Our findings were obtained from volunteering participants among the entire cohort of Berlin Police officers, who were invited to participate in 2021 and completed both lab-based tests^5^ and beSure®^15^ Critically, beSure® is the only existing bespoke tool developed for police SR selection that uses *authentic police material*, and it was specifically designed to find humans who do not make the same mistakes as an automatic solution would have (see STAR Methods and Discussion).

### Lab-based SR identification: Sensitivity, specificity, and operational implications

Our findings indicate that individuals identified as SRs via a recently proposed diagnostic framework^5^ outperformed non-SRs in the professionally relevant beSure® procedure.^15^ As a group, lab-identified SRs consistently outperformed non-SRs across all beSure® subtests. In other words, SRs demonstrated superior FIP when tested with authentic forensic materials, regardless of the stimuli presented, their viewing conditions, or tasks demands. However, multivariate and machine learning analyses revealed that these group-level differences masked substantial inter-individual differences, an increasingly acknowledged, ubiquitous feature of FIP in general.^27^^,^^28^^,^^42^ Such heterogeneity is not merely statistical noise; rather, it carries important operational implications.

First, the sensitivity of lab-based procedures appears limited; while they successfully identify individuals with professionally relevant superior ability (true positives), they also fail to detect others who performed exceptionally well on the beSure® subtests (false negatives; see Figure 2, middle and right images). Second, although lab-identified SRs were generally superior, they were not homogenous and exhibited distinct performance profiles (Figure 3). Finally, our machine learning analysis showed that beSure® subtests predicted lab-SR status with a balanced accuracy of 65%.

Taken together, these findings highlight both the value and limitations of lab-based procedures for SR identification. Such methods yield a conservative selection—minimizing false positives but *at the cost of overlooking some high-performing individuals*. As described in its technical report, the design of beSure® involved an in-depth analysis of SRs’ potential future deployment in varied tasks across the entire organization.^15^ This aligns with others’ suggestions that “[b]y understanding what tasks professional practitioners carry out, future SR tests can be tailored to more appropriately meet the requirements of these tasks.”^33^ We believe that our findings support the notion that carefully and scientifically designed, professionally tailored personnel selection solutions such as beSure® can provide ecologically relevant solutions aligned with practitioners’ operational needs, and that may deviate from procedures proposed by research(ers).

From an operational standpoint, the scientific label of “Super-Recognizer” may be less informative than an individual’s performance profile across specific, real-world tasks. For instance, an officer excelling only on the final (i.e. objectively most difficult) beSure® subtest might be particularly well suited to crowd search operations, whereas others may excel in identity verification or detecting a series among numerous uncontrolled images of unknown perpetrators. Future work - including not only a detailed account of the SR selection adopted by the Berlin Police^43^ but also longitudinal evaluation of their operational deployment - is needed to definitively assess the comparative value of lab-based vs. practitioner-designed procedures.

### Increased sensitivity via professional relevance and algorithmic complementarity

Consider the fact that a number of individuals who did not meet lab-based SR criteria excelled at beSure®. Crucially, as some participants demonstrated superior performance across not only a single test but on a selection of beSure® subtests, certainly, this observation is empirically as well as operationally meaningful. Leaving aside the fact that, as noted above, these individuals’ on-the-job performance still requires further investigation, one may ask why they exhibited different performance levels across the different SR identification tools. We believe that at least two key aspects distinguish it from lab-based procedures. Both relate to the unique stimulus material that beSure® involves—authentic forensic content derived from real police traces.

As previously noted, the beSure® subtests were deliberately designed to emulate security-relevant operational scenarios.^15^ It is conceivable that the volunteering police officers were particularly motivated to perform well on tasks they perceived as directly relevant for their professional roles. Additionally, for many of these volunteering officers, this study may have represented their first participation in an online experimental assessment. In this case, since the lab tests were completed first, the performance they measured may not have provided an accurate reflection of an individual’s underlying ability simply due to an initial lack of familiarity with behavioral FIP ability testing.

Beyond its use of authentic police materials, beSure® is distinguished by both the design and selection of its stimuli. First, its subtests were constructed to involve systematically increasing task difficulty. Second—and perhaps most critically—the selection of distractor images (i.e., non-match identities) was performed objectively using an open-source face recognition algorithm.^15^^,^^43^ Specifically, distractor images were chosen based on algorithmic false positives—images portraying faces that the algorithm incorrectly matched to the target identity. This approach represents a substantial departure from most existing lab-based FIP measures, for which researchers selected and processed the stimulus materials. By incorporating algorithmically derived distractors, beSure® identifies human observers who avoid the same classification errors as technological systems. In this way, it is inherently designed to be complementary to automated face recognition solutions.

A final consideration worth mentioning pertains to the critical differences between face recognition algorithms that exist today and that have been used previously by researchers invetsigating human and machine performance. Undoubtedly, different algorithms tend to produce different errors and, therefore, lead to different performances under different conditions. However, we contend that the most important distinction pertains to the methodology underlying a given technical solution. Perhaps, surprisingly, many existing studies have involved methods or information that are—at the least from a technical standpoint—outdated today. For instance, the Expertise in Facial Comparison Test^44^ was developed based on solutions rooted in traditional, computer vision methods.^45^^,^^46^ This contrasts with the approaches rooted in deep learning, e.g., convolutional neural networks that are commonplace today and have been used in other studies,^11^^,^^47^ and also the development of beSure®.^15^

### Limitations of the study

As described, we opted for the most conservative approach by restricting our analyses to the sample of 270 Berlin Police officers who completed “all eight tests” (three lab tests and five beSure® subtests) rather than estimating or modeling missing data points. Of this final sample, 68 met the criteria for lab-based SR identification.^5^ While our sample is large for applied face recognition research, it nonetheless limits the generalizability of our findings. Moreover, because the clustering analysis is data driven and potentially sensitive to hyperparameter selection, alternative initializations or methodologies could reveal different structures. Furthermore, our machine learning analysis did not include any hyperparameter tuning; so, it may be possible to improve on the balanced accuracy of 65%. Despite these limitations, our approach of triangulating findings through multiple complementary analyses allowed us to assess the consistency of results. Notably, we consistently observed that SRs, on average, exhibited superior performance but distinct and predictable profiles, and these differences were identified across all analyses.

### Understanding and leveraging idiosyncrasies in law enforcement

We report data obtained from the entire body of a German state police agency—a diverse cohort of individuals representing a cross-section through society. Our findings demonstrate substantial individual variability in FIP profiles across all eight behavioral measures.^1^^,^^28^^,^^42^^,^^48^^,^^49^ Importantly, these idiosyncrasies were observed not only among non-SRs, but also within the SR group. This suggests that even individuals meeting the SR diagnostic criteria cannot be expected to excel uniformly across all face-related tasks. From an operational standpoint, this underscores the importance of assessing individual ability profiles to guide personnel allocation, particularly for high-stakes police case work.

Theoretically, a deeper exploration of these performance profiles would be highly informative, for instance, to determine whether certain profile types are more prevalent at specific points along the FIP ability spectrum. However, the current dataset is not ideally suited for such an investigation. As noted above, the selection procedure and inclusion criterion (completion of all eight tests) resulted in a sample skewed toward higher-performing individuals, with approximately 25% meeting the SR criteria. Consequently, we deliberately restricted our interpretation to a descriptive level, emphasizing the presence of individual variability while deferring a detailed profile analysis to future work using a more balanced, and thus representative ability distribution.

Acknowledging and systematically leveraging these individual differences represent a promising avenue for both basic and applied research. To advance understanding of SR utility in law enforcement and judicial contexts, a number of questions warrant empirical attention. First, are SRs comparably reliable across different cases,^7^ viewing conditions,^29^^,^^30^ and time,^27^ or do specific profiles exhibit greater stability^50^? Second, to what extent do behavioral idiosyncrasies generalize to other sensory modalities or other methods (e.g., neuroimaging^51^^,^^52^^,^^53^)? Addressing these questions will lay the foundation for the *desired and necessary*, evidence-informed, human-centered improvements to the processing of facial biometric information in law enforcement.^16^

Our findings demonstrate both the value and the limits of laboratory-based identification of SRs for operational deployment in law enforcement. While lab-based diagnostic approaches^5^ may successfully identify individuals with superior FIP ability, they capture only part of the variability relevant to real-world performance. To fully exploit SRs’ naturally occurring superiority, scientifically validated, professionally relevant, role-based approaches to personnel selection and deployment are essential. In line with recent calls for greater methodological transparency and empirical validation within forensic sciences,^54^^,^^55^ we make publicly available *all data* obtained from volunteering police officers, rather than reporting or sharing only group-level summaries (e.g., Phillips et al.^8^). We hope that this large-scale investigation of professionally relevant FIP using authentic materials and procedures *co-developed with* practitioners would motivate others to follow suit in the future. Investigations of professionally relevant FIP in police contexts that can be independently reproduced, extended, and refined would help establish SRs as an invaluable human resource in modern law enforcement.^16^

## Resource availability

### Lead contact

Requests for further information and resources should be directed to and will be fulfilled by the lead contact, Meike Ramon (meike.ramon@bfh.ch).

### Materials availability

Research materials involving authentic police images (videos and still images used in beSure® subtests) cannot be shared due to their sensitive forensic nature and data protection requirements stipulated by the Data Protection Officer of the Berlin Police.

### Data and code availability

• Anonymized behavioral data reported in this paper (i.e., performance scores provided by participating police officers) and the analysis code used are publicly available from the accompanying OSF project: https://doi.org/10.17605/OSF.IO/ZWQ6B.
• Analysis code used in this paper is publicly available upon publication at the same OSF repository.
• Any additional information required to reanalyze the data reported in this paper is available from the lead contact upon request.

## Acknowledgments

M.R. thanks Simon Rjosk, the Berlin Police, in particular the Center for Innovation and Science Management, for the fruitful, long-standing collaboration and shared dedication to scientific quality and transparency, as well as all participating police officers for their support and service. This work was supported by a 10.13039/501100001711Swiss National Science Foundation PRIMA (Promoting Women in Academia) grant (PR00P1 179872) awarded to M.R. The authors thank the editor and two anonymous reviewers for their helpful feedback.

## Author contributions

M.R. designed and performed research, wrote the manuscript, and acquired funding; M.J.V. contributed new reagents/analytic tools and wrote the STAR Methods section. Both authors analyzed the data and formatted and edited the manuscript.

## Declaration of interests

M.R. acts as a Scientific Advisor to the Berlin Police; she co-developed beSure®.^15^

## STAR★Methods

### Key resources table

**Table:** 

REAGENT or RESOURCE	SOURCE	IDENTIFIER
**Deposited data**		

Anonymized behavioral data	This paper	https://doi.org/10.17605/OSF.IO/ZWQ6B

**Software and algorithms**		

Python 3.7.15	Python Software Foundation	https://www.python.org/
scikit-learn 1.0.2	Pedregosa et al.^40^	https://scikit-learn.org/
scipy 1.7.3	Virtanen et al.^56^	https://scipy.org/
R 4.2.1	R Core Team^57^	https://www.r-project.org/
ez 4.4-0	Lawrence et al.^58^	https://cran.r-project.org/package=ez
lme4 1.1-34	Bates et al.^59^	https://cran.r-project.org/package=lme4
emmeans 1.8.7	emmeans CRAN package	https://cran.r-project.org/package=emmeans
Analysis code	This paper	https://doi.org/10.17605/OSF.IO/ZWQ6B

**Other**		

Yearbook Test, long version (YBT)	Stacchi et al.^41^; Bruck, 1991^42^	
Facial Identity Card Sorting Test (FICST)	Stacchi et al.^41^; Fysh et al.^28^; Jenkins et al.^43^	
Cambridge Face Memory Test, long version (CFMT+)	Russell et al.^1^	
Berlin Test for SR Identification (beSure®)	Ramon and Rjosk^15^	

### Experimental model and study participant details

#### Human participants

The entire cohort of *N* = 17,767 Berlin Police officers employed in 2021 were invited to participate in lab-based tests for Super-Recognizer (SR) identification in April 2021; 17,681 of these were subsequently invited to participate in the first set of beSure® subtests. All methods were carried out in accordance with relevant guidelines and regulations. All experimental protocols were approved by the University of Fribourg Ethics Committee. Informed consent was obtained from all participants prior to testing.

The present analyses were restricted to participants who completed all eight sub-tests (*n* = 270; see Method Details for inclusion/exclusion criteria). Of these 270 observers, 68 met the previously proposed criteria for lab-based SR identification.^5^ Participant characteristics for the analytical sample were as follows: mean age 40.33 years (*SD* = 9.43); gender: 124 female, 146 male; handedness: 232 right-handed, 21 left-handed, 17 ambidextrous. Gender was recorded as part of the demographic registration process required prior to test access. Information on ancestry, ethnicity, and socioeconomic status was not collected, as these variables were outside the scope of the study and not part of the Berlin Police administrative data shared for research purposes.

The influence of gender on behavioral outcomes was not analyzed in the present study. The complete demographic dataset is publicly available in the deposited data (see Data and code availability). Due to the absence of gender-based analyses in the present study it is unknown whether and to what extent gender moderates the relationships between lab-based SR identification and performance on beSure®, which can be investigated based on the published data or in future work to address and improve generalizability of the findings.

### Method details

#### Study design and data inclusion

Testing was administered online via a stepwise approach, adopted to comply with the requirements of the Berlin Police Data Protection Officer (cf.^15^). Upon initial invitation, participants were required to activate their unique test battery access link and register demographic data before being presented, in random order, with the first of three lab tests. Testing phases were active for one month, followed by a one-month period during which participants could opt out and request data deletion, and then a data analysis phase.

Participants who completed all three lab tests and *underperformed* across all of them (*n* = 86) were not invited to proceed to beSure® subtests, as stipulated by the agreement with the Data Protection Officer. All other participants who completed at least one lab test were invited to beSure®. Across the stepwise approach, the number of participants gradually decreased due to this exclusion criterion and organic dropout. The present analyses were conservatively restricted to participants who completed all eight sub-tests (*n* = 270), rather than estimating or modeling missing data. No data exclusions beyond non-completion were applied. This study’s design and analyses were not pre-registered.

Of the original sample of 17,767 officers, 1,148 completed the FICST, 1,799 completed the YBT, and 1,913 completed the CFMT+, yielding an initial response rate of 9.3%. The number of participants invited to each test phase and the number who completed each test are detailed in Figure 1.

#### Lab tests for SR identification

All Berlin Police officers were first invited to complete the three well-established tests proposed by Ramon^5^ for lab-based SR identification: the Yearbook Test long version (YBT;^42^^,^^48^), the Facial Identity Card Sorting Test (FICST;^28^^,^^42^^,^^49^), and the long version of the Cambridge Face Memory Test (CFMT+;^1^). The FICST and YBT probe face *perception* across superficial image variations and considerable age-related changes in appearance. The CFMT+ is the most frequently used test of face *recognition* across increasingly difficult (degraded) visual conditions. Cut-offs for SR identification reflect previously reported normative data,^42^ applied in combination as described in Ramon.^5^

The FICST involves presentation of 40 images depicting two Dutch female-appearing celebrities. Participants are instructed to group images according to perceived identity matches. Scores are calculated as [*number of groups reported* − *total sum of within-group errors* − 2], with 0 representing a perfect score; scores of 0 or 1 are considered indicative of superior FICST performance.

The YBT comprises 8 trials (4 each for female and male identities), each involving presentation of 5 target identity images (younger appearance) alongside 10 choice options comprising the 5 target identities and 5 distractor identities showing appearance approximately 25 years older. The maximum achievable score is 35 correct; a score of 15 or higher is considered to reflect superior performance.

The CFMT+ involves experimental familiarization with 6 male-appearing target identities, which participants must identify in a 3-alternative forced-choice recognition task under increasingly difficult visual conditions (varied viewpoints, expressions, and image degradation via added noise) across 102 trials. A raw score of 85 or above is considered to reflect superior performance.

#### beSure® subtests

The subtests of beSure® were designed as increasingly challenging, ecologically valid measures of police officers’ face identity processing (FIP) ability, as detailed in Ramon and Rjosk.^15^ They probe face identity *matching* via forced-choice decisions with increasing uncertainty, implemented at three levels of chance performance: 1-to-1, 1-to-10, and 1-to-*n*. Task difficulty was further increased by systematically varying the image material: ideal, full-frontal, high-quality images versus “wild” images or videos captured under uncontrolled conditions. Across all five beSure® subtests, observers completed a total of 229 trials. Given the design of increasing task difficulty, each subtest involved a decreasing number of trials (S1: 86; S2: 70; S3 and S4: 30; S5: 12), presented randomly across two blocks of equal length.

Distractor stimuli were selected using an open-source face recognition algorithm: specifically, images of identities that the algorithm would have incorrectly matched to the target identity were chosen as distractors. Each distractor thus represents a possible algorithmic false positive. This approach ensures plausible line-ups with comparable difficulty across trials within a subtest, while also identifying human observers who avoid the same classification errors as an automated face recognition system.

### Quantification and statistical analysis

#### Software

Data were analyzed using Python version 3.7.15, with packages scikit-learn version 1.0.2^40^ and scipy version 1.7.3.^56^ Group comparison analyses were conducted using packages ez version 4.4-0,^58^ lme4 version 1.1-34,^59^ and emmeans version 1.8.7 (https://cran.r-project.org/web/packages/emmeans/emmeans.pdf) in the R environment (version 4.2.1; R Core Team, 2021).^57^

#### Group-level univariate analyses

To investigate group-level differences in beSure® performance between lab-identified SRs (*n* = 68) and non-SRs (*n* = 202), we performed a repeated-measures ANOVA with *Group* (SR vs. non-SR) as a between-participant factor and *beSure® Subtest* (S1–S5) as a within-participant factor. A Greenhouse–Geisser correction (*ϵ* ≈ 0.656) was applied to within-participant effects due to violation of sphericity (Mauchly’s test: *W* = 0.367, *p* < 0.0001). Post-hoc pairwise comparisons were Bonferroni-corrected. Effect sizes are reported as generalized eta-squared $\left(\eta_{G}^{2}\right)$ and Cohen’s *d*. Statistical parameters including exact values of *n*, means, and standard deviations are reported in the Results section and Figure 2.

#### Multivariate profile analyses

To characterize performance differences across all eight scores (three lab tests, five beSure® subtests), we computed a Performance Similarity Matrix (PSM) based on pairwise Pearson correlations between z-scored test scores across all participants, ranked by overall performance. To assess group differences in profile similarity, we extracted average within-group pairwise correlations and compared these against a surrogate null distribution generated by randomly permuting a randomly sampled submatrix of the same size as the comparison group 10,000 times. Three comparison groups of 68 observers each were examined (SRs, medium-performers, and low-performers), with a Bonferroni-corrected *α* = 0.0167 applied to account for multiple comparisons.

#### Machine learning analyses

To visualize the structure of beSure® performance profiles, we applied Uniform Manifold Approximation and Projection (UMAP;^37^) to participants’ five-dimensional subtest accuracy vectors (S1–S5). Cluster membership was determined via Hierarchical Density-Based Spatial Clustering of Applications with Noise (HDBSCAN;^38^) applied to the same accuracy profiles. Both techniques are data-adaptive and were used for exploratory visualization only.

To assess the degree to which beSure® subtest results predict lab-based SR status, we trained a Random Forest (RF) classifier^39^ using default settings as implemented in scikit-learn,^40^ which have been shown to generalize well across a range of applications.^41^ No hyperparameter tuning was performed. Model performance was estimated using Leave-One-Out Cross-Validation (LOOCV): at each iteration, all observations except one are used for training and a prediction is generated for the held-out participant; the RF is retrained from scratch at each iteration. Performance is reported as balanced accuracy to account for group size imbalance (*n*_SR_ = 68, *n*_non-SR_ = 202).
